# Poverty, malnutrition, underdevelopment and cardiovascular disease: a South African perspective

**Published:** 2007-07

**Authors:** HH Vorster, A Kruger

**Affiliations:** Africa Unit for Transdisciplinary Health Research (AUTHeR), North-West University, Potchefstroom campus, Potchefstroom; Africa Unit for Transdisciplinary Health Research (AUTHeR), North-West University, Potchefstroom campus, Potchefstroom

## Abstract

**Summary:**

This article explores possible mechanisms to explain the known relationships between poverty, undernutrition, underdevelopment and cardiovascular disease (CVD) in developing countries. Poverty is a multidimensional concept. It is both a cause and consequence of undernutrition. The article shows how malnutrition during pregnancy could lead to low birth-weight babies, who are not only at increased risk of mental and physical underdevelopment, but also ‘programmed’ to be at increased risk of CVD and other noncommunicable diseases in adult life. The underdevelopment leads to decreased ‘human capital and competence’ with an inability to create food security and an enabling environment for self and family to escape poverty and undernutrition in the next generation.

It is accepted that a lack of education and knowledge in the poor for primary prevention of CVD through healthy eating patterns and lifestyles, as well as limited access to healthcare services for secondary prevention and treatment contribute to CVD. This article postulates that the link between poverty and CVD in South Africa can be explained by the high prevalence of undernutrition in one- to nine-year-old children (9% underweight, 23% stunted and 3% wasted), the high prevalence of overweight and obesity in adults (54.5% in white men and 58.5% in African women) as well as the negative trends in nutrient intakes when Africans (the population group with the largest numbers of poor people) urbanise, acculturate and adopt westernised eating patterns that will increase CVD risk.

In conclusion, we plead for a holistic, integrated but transdisciplinary and multisectorial approach to break the vicious circle of poverty and undernutrition for the longterm prevention of CVD.

## Summary

The concept of poverty is difficult to define in simplistic terms because it is a multidimensional phenomenon with ideological and political, governance, social, economic, environmental and biological (health) components. The term poverty includes the different conditions of life that are associated with uncertainty whether essential basic needs will be met, and describes a lack of access to services and natural resources. Poverty is often characterised by a lack of freedom, education, capabilities, opportunities, employment and equity. In countries where inequity is increasing, where the gap between the rich and the poor is widening, there are often accusations that this inequity is structured and engineered by those in power and ‘the rich’.

For the purpose of this article, poverty is defined in the context of a low income of those South Africans that are in socio-economic transition, estimated in the 2003 South African Human Development Report^1^ to be almost 50% of the population. Although all four population groups include poor people, the largest numbers of poor are part of the African population group.

The relationship between poverty, undernutrition and underdevelopment has been acknowledged and understood for many years.^2^,^3^ The relationship between overnutrition and cardiovascular disease (CVD) is also well established, to the extent that both primary and secondary prevention of CVD are major motivations in the design and implementation of public health and therapeutic dietary recommendations.^4^ It is less clear how undernutrition, a consequence and cause of poverty, is associated with the increasing prevalence of CVD in developing countries – the latter documented in several sources.^5^,^6^

The objective of this article is to explore the possible mechanisms that would explain the poverty-CVD relationship in a developing country. To reach this goal, the intergenerational vicious circle between poverty, undernutrition and underdevelopment will be discussed, after which the increased risk of non-communicable diseases (NCDs) associated with foetal and infant undernutrition will be explained. The authors will then use the nutritional problems and CVD profile in South Africa to argue that malnutrition is the link between poverty and CVD.

For a variety of complex historical and political reasons, it is at present extremely difficult to disentangle the effects of socio-economic positions from racial categories in South Africa.^7^ Furthermore, the epidemiological transition from infectious diseases to NCDs experienced in middle-income countries with growing economies has been joined in South Africa by the HIV/AIDS epidemic, with a huge impact on health resources, services and health statistics,^7^ making interpretation of trends of morbidity and mortality difficult. For the purpose of this discussion, CVD refers to coronary (ischaemic) heart disease and stroke.

## The link between poverty, undernutrition and underdevelopment

**Fig. 1.**
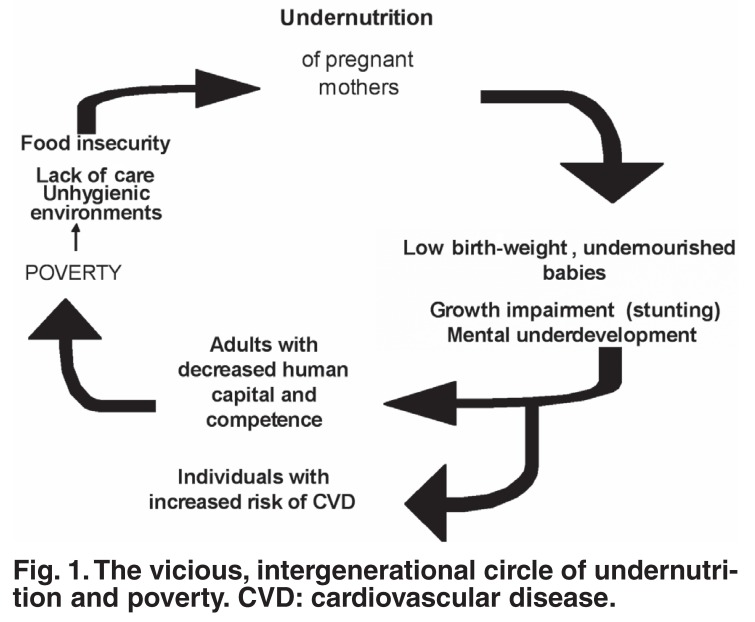
The vicious, intergenerational circle of undernutrition and poverty. CVD: cardiovascular disease.

The vicious, intergenerational circle between poverty, undernutrition and underdevelopment, illustrated in Fig. 1, is well established. The circle is ‘vicious’ because undernutrition is both a cause and a consequence of poverty.^2^,^3^ It is also ‘intergenerational’ because undernourished individuals often lack the capacity to benefit from education, to be economically productive and to create a set of circumstances that will prevent undernutrition and poverty in their offspring.

The cycle starts with malnutrition during pregnancy, which will result in low birth-weight, undernourished babies that could become stunted children and adolescents, and ‘disadvantaged’ adults when exposed to further nutritional insults during the life-cycle. These individuals have reduced physical as well as mental development and resultant lower ‘competence and human capital’. Human capital refers to well-nourished, healthy, educated, skilled and alert individuals – an improved human condition – resulting in a labour force that could be any country’s most ‘productive asset’.^8^

The Institute for Fiscal Studies^9^ describes human development as a ‘fostering of attitudes such as cognitive and noncognitive abilities and skills, nutrition and health’. They emphasise that the process begins early in infancy and continues right throughout the life of the individual. Because this process is incremental in nature, early choices, inputs and events will have the power to either debilitate or facilitate development at more advanced stages.^9^ The reduced ‘human capital’ is associated with an inability to grow optimally, develop and benefit from education; an inability to socialise, and generally a reduced capacity to lead productive lives that will ensure food security and healthy environments for self and family.

The effects of malnutrition on child development have been well researched and reported in both developed and developing world settings.^10^-^12^ Child development refers to more than only child morbidity and physical growth; it also includes behavioural–developmental aspects that promote competence. Wachs^10^ describes the competent individual as one who can effectively adapt to and interact with his or her environment. The traits defining individual competence fall into five domains: cognitive skills, temperament/personality, motivation, self-perceptions and interpersonal style. Wachs^10^ points out that these domains overlap and that the expression of individual differences in competence is partially moderated by context, including genetic, nutritional, environmental and cultural contributions.

Clearly, development of competence in infants, children and adolescents is multidetermined and needs complex, integrated interventions. There is no doubt that nutritional^11^ and other interventions^13^ that address poverty-related causes of undernutrition will improve child development, especially if they are instituted in the first three years of life.^11^ Without these interventions, malnourished children will develop into stunted, disadvantaged, ‘incompetent’ individuals who could perpetuate the undernutrition– poverty cycle.

## The link between undernutrition and CVD

Fig. 1 shows that low birth-weight babies without catch-up growth will develop into stunted children and adults with reduced human capacity. It is now recognised that foetal malnutrition not only leads to low birth-weight babies, but also to disproportionately sized babies who will have an increased risk of NCDs, including ischaemic heart disease and stroke.^14^,^15^

These ‘early origins of adult disease’ were first suspected when David Barker and his colleagues^14^ observed and documented that areas in Britain that had high rates of death from CVD used to have a high prevalence of infant mortality. Meticulous studies on individuals suffering from NCDs^15^ eventually lead to the formulation of the Barker hypothesis^14^ that ‘undernutrition *in utero* permanently changes the body’s structure and function in ways that “programme” the appearance of disease in later life’. There has been support for this hypothesis from studies all over the world, including South Africa.^16^

It is now accepted^14^-^17^ that maternal malnutrition will adversely influence the foetus, leading to, in the short term, compromised growth and a changed body composition (percentage muscle mass), changed brain development and altered metabolic programming of glucose and lipid metabolism, hormones, receptor and gene functions. In the long term, these changes will lead to reduced cognitive development, decreased educational performance, compromised immunity, lower work capacity and an increased risk of many NCDs.

There is now a better understanding of the possible underlying mechanisms responsible for the link between foetal malnutrition and risk of NCDs in later life. Gluckman and co-workers^18^ showed how malnutrition might influence genetic expression via epigenetic modification of DNA methylation in foetal life. They hypothesised that the changed genetic expression may change physiological set points that will eventually change the way individuals respond to environmental exposures in later life.

Despite these advances in our understanding of the phenomenon of the early origin of disease, much more research is needed on the programming of the various neuro-endocrine axes (and therefore regulatory potential) during foetal life. Also, more knowledge is needed on the optimal nutrition for pregnant women, optimal growth in childhood and influences of psychosocial and cultural environments, to design appropriate interventions that will minimise the risk of NCDs in adult life.

At this stage, available data show that women of childbearing age should be well nourished, empowered and educated. They are the ‘most proximal levers’^19^ to effect beneficial practices and attitudes in the home to ensure optimal foetal, infant and childhood nutrition to break the vicious circle of poverty, undernutrition, underdevelopment and NCDs.

## Poverty and CVD: evidence that malnutrition is an important link in South Africa

Bradshaw *et al.*^20^ recently showed that although the proportion of deaths from the chronic NCDs in the age group 35 to 64 years has decreased from 28.5% in 1988 to 20% in 2002 because of the increase of deaths from AIDS, the rates of deaths from CVD in all population groups are high. The all-cause ages-tandardised death rates per 100 000 in 2002 were 1 613, 937, 1 304 and 1 172 for Africans, whites, coloureds and Indians, respectively. Age-standardised CVD-related deaths per 100 000 were 375 for Africans, 384 for whites, 406 for coloureds and 607 for Indians.^20^

The same authors showed that while stroke and ischaemic heart disease are the two major causes of death in 65-year-old and older South African men and women, accounting for 30.6 and 34.9% of all deaths, HIV/AIDS, homicide and violence, and tuberculosis in the 15- to 44-year-old group accounted for 68.3% of the deaths in men and 74.2% in women. In this age group, 2.4% of the deaths in men could be attributed to stroke and ischaemic hearth disease, while 1.4% in women were caused by stroke.^20^

These analyses clearly demonstrate that CVD is a major cause of death, especially in older South Africans of all population groups, but also in younger adults. The INTERHEART Africa study^21^ showed that the CVD risk profile of black South Africans illustrates a population in the early stages of the CVD epidemic and that at this stage the risk is higher in the more educated and wealthier sector of black society, but that with growing urbanisation, rates will increase.

It can be accepted that poorer people struggling with food insecurity, lack of education and joblessness may have a lack of knowledge about CVD risk factors and little interest in primary prevention, for example, by eating low-fat, high-fibre, prudent diets. Similarly, it can be accepted that the poor will have less access to treatment and secondary prevention, especially in South Africa’s healthcare system with its limited resources, already overburdened with HIV/AIDS, tuberculosis and other infectious diseases. In addition to these two factors relating to primary and secondary prevention and treatment of CVD, there are several lines of evidence, as discussed below, that suggest that malnutrition (both under- and overnutrition) may be an important link between poverty and CVD.

Firstly, the high prevalence of undernutrition among South African children places them at risk for an increased vulnerability to NCDs in later life (see Fig. 1). The 1999 National Food Consumption study^22^ showed that among children aged one to nine years old, 9% were underweight, 23% stunted and 3% wasted: therefore, 35% of children were suffering from some form of undernutrition. Kruger et al.^23^ recently reported that stunted black children already showed a higher prevalence of increased body fat than non-stunted children. When exposed to westernised (imprudent) diets during adulthood, these children will be at an even higher risk of developing NCDs.

The second line of evidence comes from the very high rates of overweight and obesity in adult South Africans. Puoane *et al.*^24^ indicated that in the African, coloured, Indian and white groups, 25.4, 30.8, 32.7 and 54.5% of the men were either overweight or obese. The corresponding figures for women were 58.4, 52.2, 48.9 and 49.2%. Overweight and obesity are accepted risk factors for CVD, and result when the energy consumed (in the form of fat, carbohydrate, protein and alcohol) exceeds the energy needs determined by basal metabolic rate and physical activity. The high prevalence of overweight and obesity amongst African women (58.5%) of whom a large percentage are poor and suffer from food insecurity, may indicate the increased vulnerability to obesity and NCDs because of early malnutrition (as discussed in the previous section). But it also draws attention to the relationships between food insecurity, low-quality diets and obesity (large portion sizes of low micro-nutrient-dense foods).

The high prevalence of underweight in children and obesity in adults point to the co-existence of under- and overnutrition, sometimes seen in the same household in developing countries,^25^ where mothers or caregivers may be obese while the children are undernourished. This leads to the often-described double burden of undernutrition-related infections and overnutrition-related non-communicable diseases within families, communities and population groups, also experienced in South Africans. Kruger^26^ showed that the overweight and obesity of African women in the North-West Province was also related to inactivity, illustrating that not only diet, but other lifestyle factors are involved in increasing risk of NCDs.

The third line of evidence that nutrition is the link between poverty and CVD comes from the reported detrimental effects of the nutrition transition in black South Africans. When availability of natural resources (veld foods) is unlimited, rural Africans living traditional lifestyles and eating traditional diets have been shown to have a good nutritional status and very low risk factors for NCDs.^27^ However, when Africans move to urban areas, they often acculturate, modernise or westernise their eating patterns, consuming more fats and oils (more total and trans-fatty acids) more animal-derived foods (more saturated fatty acids), less staples, fruits and vegetables (less unrefined carbohydrate) and therefore often less dietary fibre and micronutrients, 28 a dietary pattern associated with an increased risk of all the NCDs, including CVD. This is another ‘double burden’: individuals ‘programmed’ during foetal life and infancy through undernutrition to be more vulnerable to CVD in later life are exposed to a modern westernised diet and nutrient intakes that will further increase risk of CVD.

## Conclusion

The major policy implication of the nutritional link between poverty and CVD is that in addition to promoting healthy lifestyles and prudent, optimal, balanced diets known to be associated with a low risk of CVD, the intergenerational cycle between poverty and undernutrition must be broken. Because poverty is both a cause and a consequence of malnutrition, any intervention should allow individuals, families and communities to realise their socio-economic rights and to have equal access to basic available resources. To eradicate malnutrition, and especially undernutrition, will only be possible if programmes address simultaneously all the immediate, underlying and basic causes of malnutrition in holistic, but integrated, multisectorial, multidisciplinary interventions that are targeted and specific (based on existing situations).

There seems to be consensus in the nutrition literature^19^ that these programmes should target women and children, with a focus on pregnant women. Furthermore, it is mandatory that food aid should not be given in isolation: it should be accompanied with education, better healthcare, creating opportunities for income generation, and creating an environment and infrastructure where the basic needs of the poor are met. Clearly, to alleviate poverty, eradicate undernutrition, and decrease the risk of CVD in adult life, a political will and commitment, a concerted effort by all involved sectors and stakeholders, as well as human and other resources are needed.
